# A Green Preconcentration Method for Determination of Cobalt and Lead in Fresh Surface and Waste Water Samples Prior to Flame Atomic Absorption Spectrometry

**DOI:** 10.1155/2012/713862

**Published:** 2012-11-22

**Authors:** Naeemullah, Tasneem Gul Kazi, Faheem Shah, Hassan Imran Afridi, Sumaira Khan, Sadaf Sadia Arian, Kapil Dev Brahman

**Affiliations:** National Centre of Excellence in Analytical Chemistry, University of Sindh, Jamshoro 76080, Pakistan

## Abstract

Cloud point extraction (CPE) has been used for the preconcentration and simultaneous determination of cobalt (Co) and lead (Pb) in fresh and wastewater samples. The extraction of analytes from aqueous samples was performed in the presence of 8-hydroxyquinoline (oxine) as a chelating agent and Triton X-114 as a nonionic surfactant. Experiments were conducted to assess the effect of different chemical variables such as pH, amounts of reagents (oxine and Triton X-114), temperature, incubation time, and sample volume. After phase separation, based on the cloud point, the surfactant-rich phase was diluted with acidic ethanol prior to its analysis by the flame atomic absorption spectrometry (FAAS). The enhancement factors 70 and 50 with detection limits of 0.26 **μ**g L^−1^ and 0.44 **μ**g L^−1^ were obtained for Co and Pb, respectively. In order to validate the developed method, a certified reference material (SRM 1643e) was analyzed and the determined values obtained were in a good agreement with the certified values. The proposed method was applied successfully to the determination of Co and Pb in a fresh surface and waste water sample.

## 1. Introduction

Release of large quantities of metals into the environment (especially in natural water) is responsible for a number of environmental problems [1]. Metals are major pollutants in marine, ground, industrial, and even treated waste waters [2]. Industrial wastes are the major source of various kinds of toxic metals which have nonbiodegradability and persistence properties resulted in a number of public health problems [3]. Metals of interest, cobalt (Co) and lead (Pb), were chosen based on their industrial applications and potential pollution impact on the environment [4].

Pb is a toxic metal and widely distributed in the environment. It is an accumulative toxic metal, which is responsible for a number of health problems [5].

Pb reaches humans from natural as well as anthropogenic sources, for example, drinking water, soils, industrial emissions, car exhaust, and contaminated food and beverages. Therefore, highly sensitive and selective methods have needed to be developed to determine the trace level of Pb in water samples. The maximum contaminant levels of Pb in drinking water allowed by environmental protection agency (EPA) is 15.0 *μ*g L^−1^, while the world health organization (WHO) for drinking water quality containing the guideline value of 10 *μ*g L^−1^ [6, 7].

Co is known to be an essential micronutrient for metabolic processes in both plants and animals [8]. It is mainly found in rocks, soil, water, plants, and animals. The determination of trace level of Co in natural waters is very important because Co is important for living species and it is part of vitamin B12 [9]. Exposures to a high level of Co lead to serious public health problems and are responsible for several diseases in human such as in lung, heart, and skin [10]. 

 Flame atomic absorption spectrometry (FAAS) is a widely used technique for quantification of metal species. The determination of metals in water samples is usually associated with a step of preconcentration of the analyte before detection [11]. The determination of trace levels of Pb and Co in water samples is particularly difficult because of the usually low concentration; on the bases of these facts a great effort is needed to develop highly sensitive and selective methods to simultaneously determine trace level of these metals in water samples [12, 13]. 

A variety of procedures for preconcentration of metals, such as solid phase extraction (SPE) [14], liquid-liquid extraction (LLE) [15], and coprecipitation and cloud point extraction (CPE) [16] have been developed. Among them, CPE is one of the most reliable and sophisticated separation methods for the enrichment of trace metals from different types of samples. While other methods such as LLE are usually time consuming and labor intensive and require relatively large volumes of solvents, which are not only responsible for public health problems but also a major cause of environmental pollution [17–22]. It was reported in literature that Pb and Co had been preconcentrated by CPE method after the formation of sparingly water-soluble complexes with different chelating agents such as ammonium pyrrolidine dithiocarbamate (APDC) [23, 24] 1-(2-thiazolylazo)-2-naphthol (TAN) [25], 1-(2-pyridylazo)-2-naphthol (PAN) [26, 27], and diethyldithiocarbamate (DDTC) [28–30].

In the present work, we introduce a simple, sensitive, selective, and low-cost procedure for simultaneous preconcentration of Co and Pb after the formation of complex with oxine, using Triton X-114 as surfactant and later analysis by flame atomic absorption spectrometry. Several experimental variables affecting the sensitivity and stability of separation/preconcentration method were investigated in detail. The proposed method was applied for the determination of trace amount of both metals in fresh surface and waste water samples. 

## 2. Experimental

### 2.1. Chemical Reagents and Glassware

 Ultrapure water, obtained from ELGA lab water system (Bucks, UK), was used throughout the work. The nonionic surfactant Triton X-114 was obtained from Sigma (St. Louis, MO, USA) and was used without further purification. Stock standard solution of Pb and Co at a concentration of 1000 *μ*g L^−1^ was obtained from the Fluka Kamica (Bush, Switzerland). Working standard solutions were obtained by appropriate dilution of the stock standard solutions before analysis. Concentrated nitric acid and hydrochloric acid were analytical reagent grade from Merck (Darmstadt, Germany) and were checked for possible trace Pb and Co contamination by preparing blanks for each procedure. The 8-hydroxyquinoline (oxine) was obtained from Merck, prepared by dissolving appropriate amount of reagent in 10 mL ethanol and diluting to 100 mL with 0.01 M acetic acid, and were kept in a refrigerator 4°C for one week. The 0.1 M acetate and phosphate buffer were used to control the pH of the solutions. The pH of the samples was adjusted to the desired pH by the addition of 0.1 mol L^−1^ HCl/NaOH solution in the buffers. For the accuracy of methodology, a certified reference material of water SRM-1643e, National Institute of Standards and Technology (NIST, Gaithersburg, MD, USA) was used. The glass and plastic wares were soaked in 10% nitric acid overnight and rinsed many times with deionized water prior to use to avoid contamination.

### 2.2. Instrumentation

A centrifuge of WIROWKA Laboratoryjna type WE-1, nr-6933 (speed range 0–6000 rpm, timer 0–60 min, 220/50 Hz, Mechanika Phecyzyjna, Poland) was used for centrifugation. The pH was measured by pH meter (720-pH meter, Metrohm). Global positioning system (iFinder GPS, Lowrance, Mexico) was used for sampling locations.

 A Perkin Elmer Model 700 (Norwalk, CT, USA) atomic absorption spectrometer, equipped with hollow cathode lamps and an air-acetylene burner. The instrumental parameters were as follows: wavelength 240.7 and 283.3 nm and slit widths: 0.2 and 0.7 nm for Co and Pb. Deuterium lamp background correction was also used.

### 2.3. Sample Collection and Preparation Procedure

The fresh surface water samples (canals) and waste water were collected on alternate month in 2011 from twenty (20) different sampling sites of Jamshoro, Sindh (southern part of Pakistan) with the help of the global positioning system (GPS). The understudy district positioned between 25° 19–26° 42 N and 67° 12–68° 02 E. The sampling network was designed to cover a wide range of the whole district. The industrial waste water samples of understudy areas were also collected. All water samples were filtered through a 0.45 micropore size membrane filter to remove suspended particulate matter and were stored at 4°C.

### 2.4. General Procedure for CPE

For Co and Pb determination, aliquot of 25 mL of the standard or sample solution containing both analytes (20–100 *μ*g/L), oxine 5 × 10^−3^ mol L^−1^ and Triton X-114 0.5% (v/v), were added. To reach the cloud point temperature, the system was allowed to stand for about 30 min into an ultrasonic bath at 50°C for 10 min. Separation of the two phases was achieved by centrifuging for 10 min at 3500 rpm. The contents of tubes were cooled down in an ice bath for 10 min. The supernatant was then decanted by inverting the tube. The surfactant-rich phase was treated with 200 *μ*L of 0.1 mol L^−1^ HNO_3_ in ethanol (1 : 1, v/v) in order to reduce its viscosity and facilitate sample handling. The final solution was introduced into the flame by conventional aspiration. Blank solution was submitted to the same procedure and measured in parallel to the standards and real samples. 

## 3. Result and Discussion

### 3.1. Optimization of CPE

 The preconcentration of Pb and Co was based on the formation of a neutral, hydrophobic complex with oxine, which is subsequently trapped in the micellar phase of a nonionic surfactant (Triton X-114). Utilizing the thermally induced phase extraction separation process known as CPE, the analyte is highly preconcentrated and free of interferences in a very small micellar phase. Several parameters play a significant role in the performance of the surfactant system that is used and its ability to aggregate, thus entrapping the analyte species. The pH, complexing reagent and surfactant concentration, temperature, and time were studied for optimum analytical signals.

### 3.2. Effect of pH

**Figure 1:**
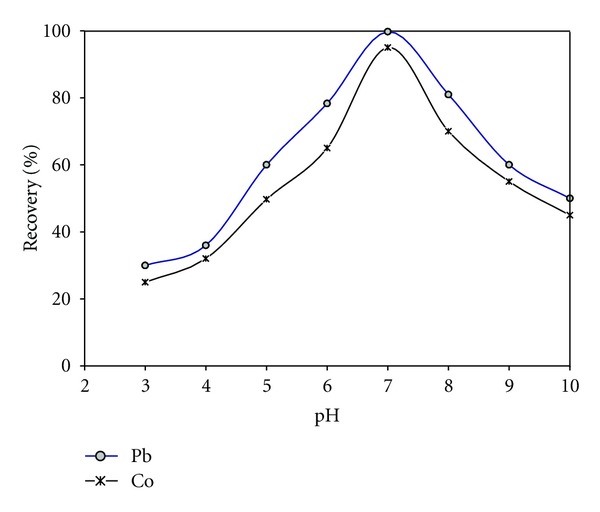
Effect of pH on the percentage of recovery: 20 *μ*g L^−1^ of Pb and Co, 5.0 × 10^−3^ mol L^−1^ oxine, 0.5% (v/v) Triton X-114, temperature 50°C, and centrifugation time 10 min (3500 rpm).

The effect of pH on the CPE of Co and Pb was investigated because this parameter plays an important role in metal-chelate formation. The effect of pH upon the extraction of Co and Pb ions from the six replicate standard solutions 20.0 *μ*g L^−1^ was studied within the pH range of 3–10, while each operational desired pH value was obtained by the addition of 0.1 mol L^−1^ of HNO_3_/NaOH in the presence of acetate/borate buffer. The maximum extraction efficiency of understudy metals was obtained at pH range of 6.5–7.5 as shown in Figure 1, for subsequent work pH 7.0 was chosen as the optimum for subsequent work.

### 3.3. Effect of Triton X-114 Concentration

**Figure 2:**
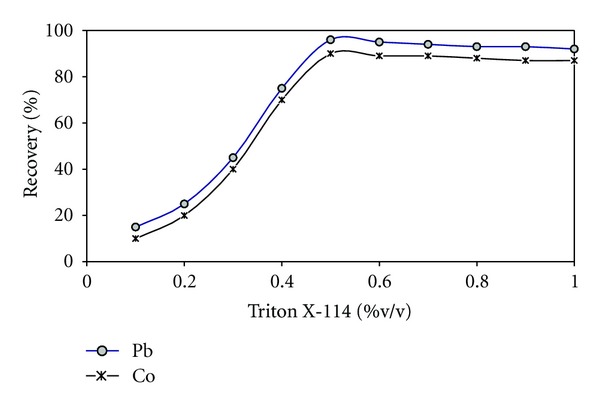
Effect of Triton X-114 on the percentage of recovery: 20 *μ*g L^−1^ of Pb and Co, 5.0 × 10^−3^ mol L^−1^ oxine, pH 7.0, temperature 50°C, and centrifugation time 10 min (3500 rpm).

Separation of metal ions by a cloud point method involves the prior formation of a complex with sufficient hydrophobicity to be extracted in a small volume of surfactant-rich phase. The temperature corresponding to cloud point is correlated with the hydrophilic property of surfactants. The nonionic surfactant Triton X-114 was chosen as surfactant due to its low cloud point temperature and high density of the surfactant-rich phase, which facilitates phase separation by centrifugation. The effect of Triton X-114 concentrations on the extraction efficiencies of Co and Pb were examined at the range of 0.1 to 1.0% (v/v).  Figure 2 shows that quantitative extraction was observed when surfactant concentration was > 0.5% (v/v). At lower concentrations, the extraction efficiency of complexes was low probably because of the inadequacy of the assemblies to entrap the hydrophobic complex quantitatively. A Triton X-114 concentration of 0.5% (v/v) was selected for subsequent studies.

### 3.4. Effect of Oxine Concentration

**Figure 3:**
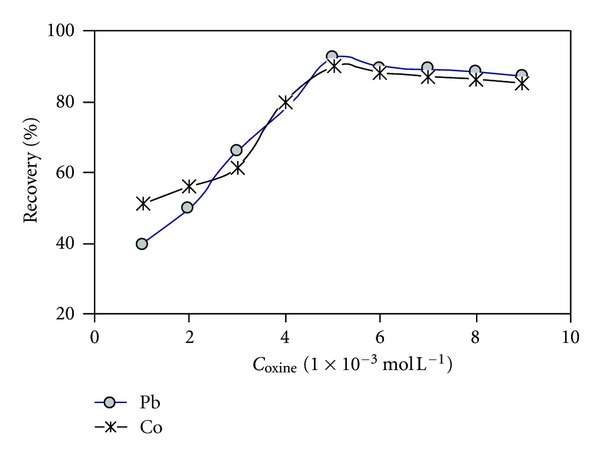
Effect of oxine concentration on the percentage of recovery: 20 *μ*g L^−1^ of Pb and Co, 0.5% (v/v) Triton X-114, pH 7.0, temperature 50°C, and centrifugation time 10 min (3500 rpm).

 The oxine is a relatively very stable and selective hydrophobic complexing reagent which reacts with both selected cations. Replicate 10 mL of standard, SRM, and real sample solution in 0.5% (w/v) Triton X-114 at a buffer of pH 7.0 and complexed with oxine solutions in the range of 1.0–10.0 × 10^−3 ^mol L^−1^. The results revealed in Figure 3 that extraction efficiency of both metals increases up to 5 × 10^−3 ^mol L^−1^. This value was, therefore, selected as the optimal chelating agent concentration. The concentrations above this value have no significant effect on the efficiency of CPE.

### 3.5. Effects of Sample Volume on Preconcentration Factor

The preconcentration factor (PCF) is defined as the concentration ratio of the analyte in the final diluted surfactant-rich extract ready for its determination and in the initial solution. Among the other factors, this depends on the phase relationship, on the distribution constant of the analyte between the phases, and on sample volume. The sample volume is one of the most important parameters in the development of the preconcentration method, since it determines the sensitivity and enhancement of the technique. The phase ratio is an important factor, which has an effect on the extraction recovery of cations. A low phases ratio improves the recovery of analytes, but decreases the preconcentration factor. However, to determine the optimum amount of the phase ratio, different volumes of a water sample 10–1000 mL and a constant volume of surfactant solution 0.5% were chosen. The obtained results show that with increasing the sample volume >100 mL, the extracted understudy analytes were decreased as compared to those obtained with 25–50 mL. A successful cloud point extraction should maximize the extraction efficiency by minimizing the phase volume ratio, thus improving its concentration factor. In the present work, the initial sample volume was 25 mL and the final volume of surfactant rich phase after diluted with acidic ethanol was 0.5 mL, hence the PCF achieved in this work was 50 for both understudy analytes. 

### 3.6. Interferences

The interference is those relating to the preconcentration step, which may react with oxine and decrease the extraction. To perform this study, 25 mL solution containing 20 *μ*g/L^−1^ of both metals at different interference to analyte ratio were subjected to the developed procedure.  Table 1 shows the tolerance limits of the interfering ions error <5%. The tolerance limit of coexisting ions is defined as the largest amount making variation of less than 5% in the recovery of analytes. The effects of representative potential interfering species were tested. Commonly encountered matrix components such as alkali and alkaline earth elements generally do not form stable complexes under the experimental conditions. A high concentration of oxine reagent was used, for the complete chelation of the selected ions even in the presence of interferent ions.

### 3.7. Analytical Figures of Merit

The calibration graph using the preconcentration step for Co and Pb were linear with a concentration range of 5.0–20 ug L^−1^ of standards and subjecting to CPE methods at optimum levels of all understudy variables. The extracted analytes in diluted micellar media were introduced into the flame by conventional aspiration.  Table 2 gives the calibration parameters for the proposed CPE method including the linear ranges, relative standard deviation RSD, and limit of detection LOD. The experimental enhancement factors calculated as the ratio of the slopes of calibration graphs with and without preconcentration. The enhancement factors of Co and Pb subjected to CPE method were found to be 70 and 50, respectively. The limits of detection LOD were calculated as the ratio between three times the standard deviation of ten blank readings and the slope of the calibration curve after preconcentration were calculated as 0.26 and 0.44 *μ*g L^−1^, respectively, for Co and Pb. The obtained LOD was sufficiently low for detecting trace levels of Co and Pb in different types of fresh and waste water samples. 

The accuracy of the proposed method was evaluated by analyzing a standard reference material of water SRM-1643e with certified values of Co and Pb content. It was found that there is no significant difference between results obtained by the proposed method and the certified results of both metals. Reliability of the proposed method was also checked by spiking both metals at to three concentration levels (2.0–10.0 *μ*g L^−1^) in a real water sample. The results are presented in Tables 3(a) and 3(b). The perecentage of recoveries (*R*) of spike standards were calculated as follows:

(1) $$\begin{array}{c} R\;\left(\%\right)=\frac{\left(C_{m}-C_{\text{o}}\right)}{m}\times 100, \end{array}$$

where *C*_*m*_ is a value of metal in a spiked sample, *C*_o_ the value of metal in a sample, and *m* is the amount of metal spiked. These results demonstrate the applicability of developed procedure for Co and Pb determination in different water samples. 

### 3.8. Application to Real Samples

The CPE procedure was applied to determine Co and Pb in fresh surface and waste water samples. The results are shown in Table 4. The Co and Pb concentrations in fresh surface water were found in the range of 2.12–5.12 *μ*g L^−1^ and 1.49–8.56 *μ*g L^−1^, respectively. In waste water, the levels of both analyte were high, found in the range of 13.6–16.8 and 15.1–19.4 *μ*g L^−1^ for Co and Pb, respectively.

## 4. Conclusion

 In this study, Triton X-114 was chosen for the formation of the surfactant-rich phase due to its excellent physicochemical characteristics, low cloud point temperature, high density of the surfactant-rich phase, which facilitates phase separation easily by centrifugation, and commercial availability and relatively low price and low toxicity. This method is a promising alternative for the determination of Co and Pb linked with FAAS. From the results obtained, it can be considered that oxine is an efficient ligand for cloud point extraction of Co and Pb. The simple accessibility, the formation of stable complexes, and consistency with the cloud point extraction method are the major advantages of the use of oxine in cloud point extraction of Co and Pb. CPE has been shown to be a practicable and versatile method, being adequate for environmental studies. Cloud point extraction is an easy, safe, rapid, inexpensive, and environmentally friendly methodology for preconcentration and separation of trace metals in aqueous solutions. The surfactant-rich phase can be directly introduced into flame atomic absorption spectrometer FAAS after dilution with acidic ethanol. The proposed CPE method incorporating oxine as chelating agent permits effective separation and preconcentration of Co and Pb and final determination by FAAS provides a novel route for trace determination of these metals in water samples of different ecosystem. A low-cost surfactant was used, thus toxic organic solvent extraction generating waste disposal problems was avoided. The comparison of the results found in the presented study and some works in the literature was given in [31–44]. The proposed cloud point extraction method is superior for having lower detection limits when compared to other methods as shown in Table 5.

## Figures and Tables

**Table 1:** Influences of some foreign ions on the recoveries of cobalt and lead (20 *μ*g L^−1^) determination by applied CPE method.

Ion	Concentration (*μ*g L^−1^)	Pb recovery (%)	Co recovery (%)
Na^+^	20000	97 ± 2.14	98 ± 2.22
K^+^	5000	98 ± 2.21	99 ± 3.02
Ca^2+^	5000	98 ± 2.12	97 ± 1.12
Mg^2+^	5000	97 ± 1.04	98 ± 3.08
Cl^−^	30000	99 ± 2.05	98 ± 3.04
F^−^	1000	96 ± 3.01	97 ± 1.12
NO^3−^	3000	97 ± 1.04	98 ± 3.06
HCO_3_^−^	1000	98 ± 3.12	97 ± 2.04
Al^3+^	500	97 ± 2.21	99 ± 3.05
Fe^3+^	50	96 ± 2.23	97 ± 3.02
Zn^2+^	100	97 ± 3.05	96 ± 2.32
Cr^3+^	100	96 ± 2.08	98 ± 2.25
Cd^2+^	100	97 ± 3.12	98 ± 3.22
Ni^2+^	100	96 ± 2.23	97 ± 3.01

**Table 2:** Analytical characteristics of the proposed method.

Element condition	Concentration range (*μ*g L^−1^)	Slope	Intercept	*R* ^2^	R.S.D. (*n* = 5)^a^	LOD^b ^(*μ*g L^−1^)
Co without preconcentration	250–5000	3.97 × 10^−3^	−0.013	0.9871	1.45 (500)	32.0
Co with preconcentration	20.0–100	0.279	+0.008	0.9997	2.22 (20)	0.26
Pb without preconcentration	250–5000	5.03 × 10^−3^	−0.034	0.9972	0.88 (600)	46.0
Pb with preconcentration	20.0–100	0.256	−0.012	0.9989	1.88 (30)	0.44

^a^Values in parentheses are the Co and Pb concentrations (*μ*g L^−1^) for which the RSD was obtained.

^b^Limit of detection, calculated as three times the standard deviation of the blank signal.

**Table:** (a)

Certified reference material	Certified values (*μ*g L^−1^)		Measured values (*μ*g L^−1^)		Percentage of recovery (RSD %)	
	Co	Pb	Co	Pb	Co	Pb
SRM 1643e	27.06 ± 0.3	19.63 ± 0.2	26.8 ± 0.82	19.24 ± 0.5	99.0% (3.06%)	98.0% (2.60%)

**Table:** (b)

Samples	Added (*μ*g L^−1^)		Measured (*μ*g L^−1^)		Recovery (%)	
	Co	Pb	Co	Pb	Co	Pb
Canal water	0	0	3.34 ± 0.962	6.08 ± 0.781	—	—
	2	2	5.32 ± 0.384	8.06 ± 0.822	99.8	99
	5	5	8.33 ± 0.432	11.0 ± 0.784	100	98.4
	10	10	13.3 ± 0.642	15.9 ± 0.828	99.6	98.2

Mean ± SD (*n* = 3).

**Table 4:** Determination of lead and cobalt in water samples.

Sample	Co (*μ*g L^−1^)	Pb (*μ*g L^−1^)
Canal water	3.34 ± 0.962	6.08 ± 0.781
Waste water	14.6 ± 1.20	17.3 ± 1.52

Mean ± SD (*n* = 3).

**Table 5:** Comparative table for determination of cobalt and lead in different types of samples applying CPE before analysis by atomic spectrometric technique.

Reagent and surfactant	Matrix and technique	PF^a^ and EF^b^	LOD^c ^(*μ*g L^−1^)	Reference
Cobalt				

TAN/Triton X-114	Water/(FAAS)	**—/**57^b^	0.24	[25]
PAN/TX-100	Water samples/(GFAAS)	**—/**100^b^	0.003	[31]
PAN/TX-114	Urine/(FAAS)	**—/**115^b^	0.38	[32]
5-Br-PADAP/TX-100-SDS	Pharmaceutical samples/(FAAS)	**—**/29^b^	1.1	[14]
TAN/Triton X-100	Water/(GFAAS)	**—**/100^b^	0.003	[33]
APDC/Triton X-114	Biological tissues/(TS-FF-FAAS)	**—**/130^b^	2.1	[34]
APDC/Triton X-114	Water/(FAAS)	**—**/20^b^	5.0	[23]
1,2-N,N /PONPE 7.5	Water sample/(FAAS)	**—**/27^b^	1.22	[35]
Me-BTABr/Triton X-114	Water sample/(FAAS)	**—**/28^b^	0.9	[36]
Oxine/Triton X-114	Water sample/(FAAS)	50/50	0.44	Present work

Lead				

DDTP/Triton X-114	Human hair/(FAAS)	**—**/43^b^	2.86	[28]
PONPE 7.5/—	Human saliva/(FAAS)	**—**/10^b^	—	[37]
APDC/Triton X-114	Certified biological reference materials/(ETAAS)	**—**/22.5^b^	0.04^c^/—	[24]
DDTP/Triton X-114	Certified blood reference samples/(ETAAS)	**—**/34^b^	0.08	[38]
PONPE 7.5/—	Tap water certified reference material/(ICP-OES)	**—**/>300^b^	0.07	[39]
DDTP/Triton X-114	Riverine and sea water enriched water reference materials/(ICP-MS)	**—/—**	40.0	[40]
5-Br-PADAP/Triton X-114	Water/(GFAAS)	50^a^/**—**	0.08^c^/—	[41]
PAN/Triton X-114	Water/(FAAS)	**—/**55.6^b^	1.1	[27]
—/Tween 80	Environmental sample/FAAS	10^a^/**—**	7.2	[42]
TAN/Triton X-114	Water sample/(FAAS)	15.1^a^/**—**	4.5	[43]
Pyrogallol/Triton X-114	Water sample/(FAAS)	72^a^/**—**	0.4	[44]
Oxine/Triton X-114	Water sample/(FAAS)	50/70	0.26	Present work

^a^preconcentration factor, ^b^enhancement factor, and ^c^limit of detection.
